# Experiences, challenges, and best practices of dispatcher-assisted cardiopulmonary resuscitation: a scoping review

**DOI:** 10.1007/s11739-025-03991-7

**Published:** 2025-08-11

**Authors:** Guglielmo Imbriaco, Alessandro Galazzi, Federico Semeraro, Nicola Ramacciati

**Affiliations:** 1 Department of Biomedicine and Prevention, University of Rome “Tor Vergata”, Rome, Italy; 2 118 Emilia Est Emergency Medical Communication Center, Maggiore Hospital Carlo Alberto Pizzardi, Largo Bartolo Nigrisoli 2, 40133 Bologna, Italy; 3 Department of Medicine and Surgery, LUM University, Strada Statale 100, Km 18, 70010 Casamassima, Bari Italy; 4 Department of Anesthesia, Intensive Care and Prehospital Emergency, Maggiore Hospital Carlo Alberto Pizzardi, Bologna, Italy; 5 Department of Pharmacy and Health and Nutrition Sciences, University of Calabria, Cosenza, Italy

**Keywords:** Dispatcher-assisted cardiopulmonary resuscitation, Emergency medical communication centre, Out-of-hospital cardiac arrest, Scoping review, Telephone CPR

## Abstract

**Background:**

Out-of-hospital cardiac arrest is a leading cause of mortality, with survival rates from 8 to 13%. Dispatcher-assisted cardiopulmonary resuscitation (DA-CPR) may increase bystander-initiated CPR, improving survival outcomes. Despite these benefits, DA-CPR is often hindered by barriers and organizational issues.

**Objective:**

To map and summarize the existing literature on DA-CPR, to identify strategies to improve provision rates, overcoming potential barriers.

**Eligibility criteria:**

Primary studies with an English abstract, published between 2018 and 2024, focusing on experiences, challenges, and best practices related to DA-CPR. Studies were included if they reported on emergency callers and dispatchers (population), focusing on DA-CPR provision methods (concept), in any setting (context). Simulation studies were excluded.

**Sources of evidence:**

The following databases were searched: Medline (PubMed), EMBASE, CINAHL, and the Cochrane Library. Grey literature from repositories, conference proceedings, and social media were also reviewed.

**Charting methods:**

Characteristics of the included studies were reported in a specific extraction table and synthesized narratively.

**Results:**

Fifty-eight studies were included. Results were categorized into themes (dispatcher, caller, system, and community/context). Dispatcher training, simplified protocols, effective communication, and video calls emerged as elements potentially improving DA-CPR provision. Caller-related barriers like emotional distress and language problems were prevalent. System-level interventions, including centralized call-handling and performance evaluations, improved DA-CPR rates. Community initiatives for CPR education enhanced bystander compliance.

**Conclusions:**

This scoping review identifies strategies to enhance DA-CPR provision, emphasizing tailored dispatcher protocols, communication strategies, system-level improvements, and community-based interventions. Future research should evaluate the effectiveness of these strategies to optimize out-of-hospital cardiac arrest response.

**Supplementary Information:**

The online version contains supplementary material available at 10.1007/s11739-025-03991-7.

## Introduction

Out-Of-Hospital Cardiac arrest (OHCA) is a leading cause of mortality, with survival rates ranging from 8–13% [1–3]. Early Cardiopulmonary Resuscitation (CPR) is crucial, as survival decreases by 7–10% for each minute without intervention [4]. While waiting for the arrival of Emergency Medical Service (EMS) teams, Bystander-CPR (B-CPR) can double or triple survival and improve neurological outcomes; despite these advantages, it is performed in only 58% (13–83%) of cases on average [5].

Improved survival following OHCA has been linked to community-based interventions, such as the rapid initiation of B-CPR and the availability of Automated External Defibrillators (AEDs) [6]. The role of Emergency Medical Communication Centre (EMCC) dispatchers emerges as a key feature of the “Systems Saving Lives” concept, proposed by the European Resuscitation Council (ERC) in 2021, outlining that their responsibilities extend beyond call handling, including the rapid recognition of OHCAs and the provision of CPR instructions [4]. This framework recognizes that survival is dependent on a well-coordinated system involving multiple components: prompt identification of OHCA and early activation of EMS, B-CPR with or without dispatchers’ support, public education and awareness initiatives on CPR, public access defibrillation, and specialized post-resuscitation care in cardiac arrest (CA) centers [4].

Over the last decade, dispatcher-assisted cardiopulmonary resuscitation (DA-CPR), also known as telecommunicator-CPR or Telephone-CPR (T-CPR), has been increasingly implemented to enhance early bystander intervention during OHCA [7, 8]. However, DA-CPR uptake remains suboptimal, with refusal rates between 23.5% and 50.3% due to physical, emotional, or language barriers [9–11].

Although DA-CPR has been practiced for over 30 years [12], its formal role was first highlighted in CPR guidelines after 2010, gaining greater emphasis in subsequent updates [13, 14]. Despite scientific recommendations [15], many EMCCs, especially in low-resource countries, still lack formal DA-CPR programs or standardized performance measures [16–18]. Emerging technologies like video calls present new opportunities to strengthen interactions between dispatchers and bystanders [19, 20].

Although several reviews explored DA-CPR [21–23], relatively few provide a comprehensive synthesis of the interactions between dispatchers, callers, EMCC systems, and the community; moreover, key aspects (dispatchers' background, training and retraining, instruction sequencing, quality improvement components, and the integration of new technologies) remain insufficiently understood [24]. Furthermore, recent research addressing interventions to optimize DA-CPR found that almost half of the available evidence was performed in simulated settings, calling for a better understanding of facilitators and barriers, exploring real-world OHCA events [25, 26]. Albeit simulation-based research provides valuable insights into training effectiveness and decision-making processes, it may not fully reflect the complexities and challenges of real calls, including caller distress, communication barriers, and variations in real-life dispatcher performance [25, 27].

Owing to the complex clinical, operational, and communication aspects of DA-CPR, the scoping review methodology is the most appropriate for synthesizing diverse evidence, allowing for a broader exploration of the available literature and identifying knowledge gaps [28].

## Objective

This scoping review aims to explore and synthesize DA-CPR literature, focusing on real-world experiences, challenges, and best practices, to identify strategies improving DA-CPR rates and overcoming potential barriers. The following review questions were used: (1) What are the best practices for dispatchers when giving CPR instructions? (2) What challenges and barriers can arise when providing DA-CPR? (3) What strategies can help promote the implementation of DA-CPR?

## Methods

### Design

This scoping review followed the Joanna Briggs Institute (JBI) methodology [28] and is reported according to the Preferred Reporting Items for Systematic Reviews and Meta-Analyses extension for Scoping Reviews (PRISMA-ScR) checklist (Supplementary Material 1) [29]. The protocol was previously published [30].

### Eligibility criteria

This scoping review examined experiences, challenges, and best practices of DA-CPR, researching strategies and organizational factors influencing their provision. During the literature analysis, the authors noted that CA outcomes are affected by factors beyond DA-CPR, including comorbidities, response times, and bystander actions. Aligning with Aldridge et al. [10], the Population, Concept, and Context (PCC) was:

- Population: EMCC dispatchers and OHCA witnesses who call EMS, as both play key roles in the DA-CPR process;
- Concept: The provision of DA-CPR, analyzing specific experiences and challenges at the system, provider, and bystander levels;
- Context: Real-world settings, regardless of geographic location, including consideration of sociocultural factors, resource availability, and systemic differences.

We included primary research articles, published between 2018 and 2024, with at least an English abstract, that reported real-world DA-CPR experiences. Full inclusion and exclusion criteria are provided in Table 1. The 2018–2024 timeframe was chosen to provide an overview of current international DA-CPR practices, including studies conducted after the 2015 resuscitation guidelines and addressing gaps identified in the 2019 COSTR [24, 31]. While Nikolaou et al. reviewed survival outcomes up to mid-2018 [21], we focused instead on the real-world implementation of DA-CPR, excluding simulation studies to prioritize practical experiences. The evolving role of simulation in CPR has been specifically addressed in other studies [27, 32].

**Table 1:** Inclusion and exclusion criteria for study selection

Inclusion criteria	Rationale
Primary studies conducted on DA-CPR, involving human subjects of any age All study designs, including experimental and observational studies Peer-reviewed journal articles Conference papers and other grey literature Published studies with an English abstract Studies published between 2018 and 2024	To provide a comprehensive analysis of DA-CPR across different population groups To capture a broad spectrum of evidence, from real-world experiences To ensure methodological quality and reliability To increase the number of reported experiences on DA-CPR and provide insights into ongoing research and emerging trends To provide comprehensive information for initial eligibility assessment To capture studies and real-world experiences conducted after the introduction of the 2015 resuscitation guidelines and to address knowledge gaps highlighted in the 2019 international CoSTR
**Exclusion criteria**	**Rationale**
Research focusing solely on bystander CPR without dispatcher involvement Simulation-based studies Secondary research articles (e.g., systematic reviews and meta-analyses)	To exclude interventions not aligned with the research objectives To focus on real-world dispatcher interventions rather than controlled training environments To analyze primary data from original research

*CoSTR* Consensus on Cardiopulmonary Resuscitation and Emergency Cardiovascular Care Science with Treatment Recommendations, *DA-CPR* dispatcher-assisted cardiopulmonary resuscitation

### Search strategy and information sources

The search was performed in four databases: Medline (PubMed), EMBASE, CINAHL, and the Cochrane Library for articles indexed between January 1, 2018, and December 31, 2024. A filter for human studies was applied to exclude simulated studies and studies on animal models, ensuring that only research involving real human subjects was considered. Reference lists of relevant articles were also screened. Search strings are provided in Supplementary Material 2.

The EMBASE search yielded several conference abstracts and posters, all included for screening. Additional grey literature searches were conducted in Scopus for conference proceedings and the Open Access Theses and Dissertations (OATD) portal for theses and dissertations, using the same keywords and timeframe. When a thesis was accompanied by a peer-reviewed publication, only the article was included.

In addition, we screened the social media LinkedIn and Twitter/X using the terms “dispatcher-assisted CPR”, “dispatcher-assisted cardiopulmonary resuscitation”, “telephone CPR”, and “telephone cardiopulmonary resuscitation”. Posts and threads in the timeframe 2018–2024 were reviewed for information reporting real-world experiences, training programs, or system-level practices related to DA-CPR. These sources were screened by one reviewer, and if deemed potentially relevant to the research questions, were discussed with an additional reviewer before inclusion.

### Selection of sources of evidence

Retrieved records were uploaded to the online software Rayyan [33]. After duplicate removal, two independent researchers screened titles and abstracts for inclusion and assessed the full texts of relevant sources. Disagreements during the selection process were resolved through discussion, with a third researcher consulted to achieve consensus.

### Data charting process and synthesis of results

The authors developed a data-charting form de novo using Microsoft Excel to extract and organize key information on author, publication year, country, study design, objectives, relevance for PCC, and interventions or experiences, specifically advantages (“pros”) and challenges or limitations (“cons”), related to DA-CPR.

One researcher performed the initial data extraction, which was independently verified by a second researcher; any discrepancies were resolved by consulting a third researcher. The results were synthesized narratively and categorized into four main categories according to the PCC (dispatcher, caller, system, and community/context) and further organized into subcategories, providing a more detailed description of the interventions. Some papers spanned multiple themes and were accordingly referenced across categories to preserve the complexity and multidimensionality of their results. Finally, the synthesized information was reviewed by three expert EMCC dispatchers and two members of a resuscitation scientific society to ensure accuracy and practical relevance.

### Critical appraisal of sources of evidence

According to JBI recommendations, critical appraisal or risk of bias assessment were not performed, as the primary aim of scoping reviews is to map the extent and nature of the available literature [28].

## Results

The database search yielded 629 records. After removing duplicates, 441 records were screened by title and abstract. Of these, 324 were excluded for not meeting the inclusion criteria. The remaining 117 full-text documents were assessed for eligibility. At this stage, 59 documents were excluded: not relevant (*n* = 53), not aligned with PCC (*n* = 2), wrong publication type (*n* = 2), and conference abstracts with data reported in an included full article (*n* = 2). Ultimately, 58 documents met the inclusion criteria and were included in the final analysis. No additional papers were identified from reference lists.

**Fig. 1:**
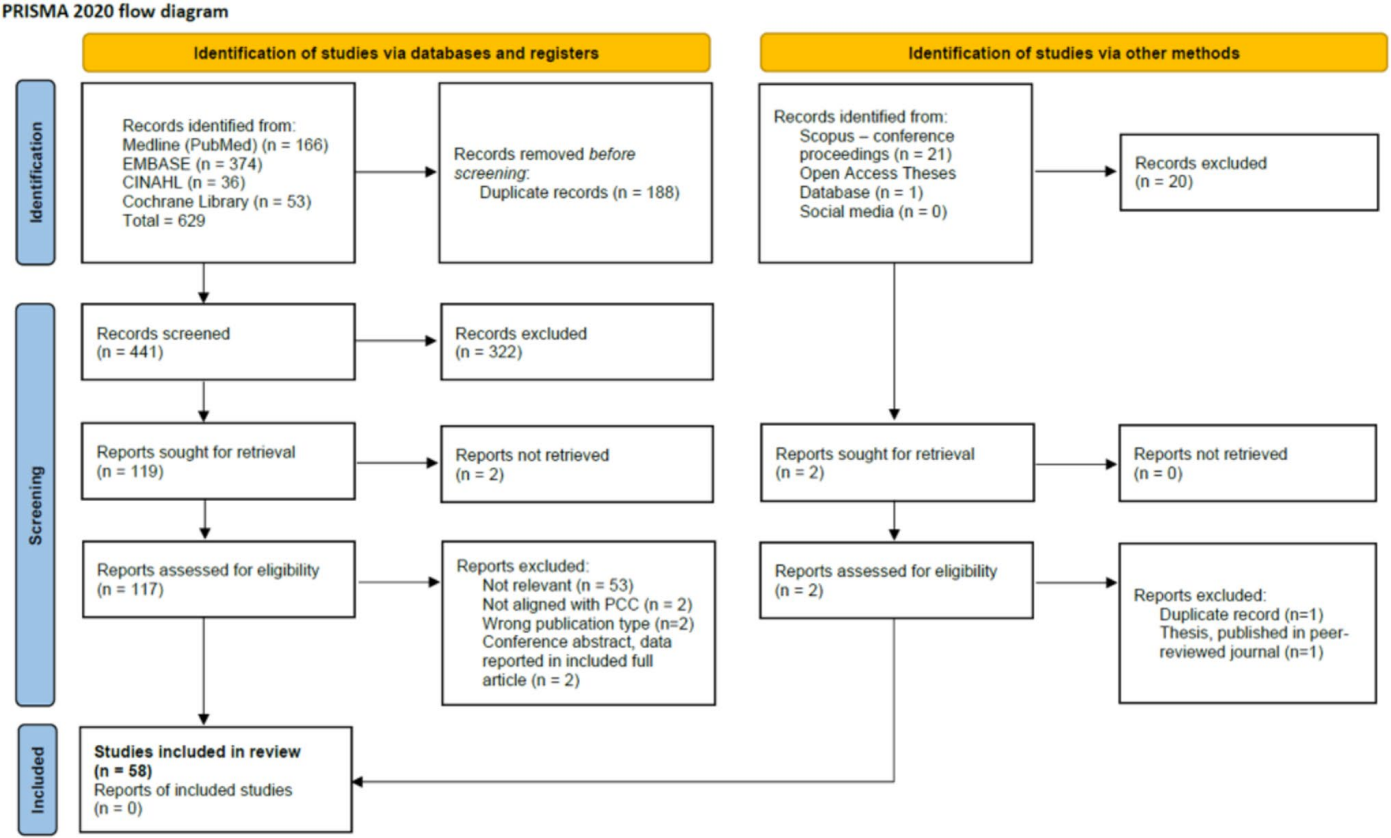
PRISMA flow diagram

The grey literature search retrieved 21 conference papers from Scopus, but the only one relevant was already included. One thesis from OATD was excluded in favor of its peer-reviewed publication. No social media posts met the inclusion criteria and were excluded from the final review. The screening and selection process is shown in the PRISMA flow diagram (Fig. 1) [34].

The included studies were published from 2018 to 2024. A majority of studies (*n* = 39, 67.2%) originated from Asia [10, 11, 17, 18, 35–69], North America accounted for 9 studies [70–78], Europe for 6 [16, 79–83], and Oceania for 4 (all from Australia) [84–87]. Most included studies (*n* = 30, 51.7%) had a retrospective cohort design [10, 11, 17, 39, 41, 43, 44, 46, 48, 52, 54–56, 62–67, 70, 71, 74, 76, 77, 79, 80, 84–87]; 7 were conference papers [46, 49, 54, 55, 72, 78, 80]. Study characteristics are summarized in Table 2.

**Table 2:** Characteristics of the included studies

Authors Publication year	Country	Aim	Design	PCC	Pros	Cons
Riou et al. 2018	Australia	To examine whether the language used by dispatchers to initiate CPR had an impact on callers’ agreement to perform CPR	Retrospective cohort	Dispatcher	Talking in terms of futurity in the CPR opening was associated with high caller agreement to perform CPR	Talking in terms of willingness in the CPR opening was associated with low caller agreement to perform
Yamashita et al. 2018	Japan	To investigate temporal variations in DA-CPR	Retrospective cohort	Community/context	Alerting systems to recruit well-trained neighboring individuals may improve the survival of OHCAs witnessed by bystanders other than family during night time	Low DA-CPR rate during night-time (probably related to EMCC staffing)
Nagao et al. 2018	Japan	To compare DA-CPR instruction according to 2005 and 2010 guidelines—simplified algorithm (NO look-listen-feel, chest compression-only if not CPR trained)	Retrospective cohort *	Dispatcher	Higher DA-CPR rate with 2010 recommendations (simplified algorithm + chest compression only)	
Ko et al. 2018	South Korea	To evaluate whether the effect of the detection time interval (DTI) on neurological outcome after DA-CPR were significantly different between the urbanization levels	Cross-sectional	Dispatcher, system	A simpler and easier DA-CPR protocol based on two criteria (altered mental status and abnormal breathing) may reduce DTI	Agonal breathing and misinterpretation of seizures can delay OHCA detection. Large call volumes (e.g. highly urbanized areas) increase times, particularly in case of two-tier EMCC systems
Shah et al. 2018	USA	1) To compare the current dispatch practice to proposed DA-CPR guidelines; 2) To describe physical and logistical barriers to DA-CPR provision; 3) To assess the association of DA-CPR with OHCA survival to hospital discharge	Retrospective cohort	Dispatcher, caller, system	DA-CPR instructions were associated with a substantially increased rate of CPR provision. Operational and logistical barriers, in addition to cognitive barriers, must be evaluated and identified	The primary barriers to DA-CPR are the failure to transfer the call to a DA-CPR capable dispatcher and the inability of callers to move a patient to perform CPR
Chang et al. 2018	South Korea	To demonstrate the association of B-CPR with survival outcomes after pediatric OHCA by community property value groups	Cross-sectional	Caller, Community/context	Tailored DA-CPR programs and CPR training strategies targeting lower socioeconomic groups are needed to enhance compliance of laypersons and to provide effective CPR for pediatric OHCA that occur in neighborhoods with relatively low wealth	
Ro et al. 2018	South Korea	To evaluate the associations between the centralization of dispatch centers and DA-CPR for OHCA	Prospective observational	System	Increasing dispatchers’ experience with OHCAs by concentrating emergency calls may enhance bystander CPR and DA-CPR instructions for OHCA patients. Centralization of dispatch centers could be one strategy for delivering effective DA-CPR instructions and increasing bystander CPR	
Case et al. 2018	Australia	To identify barriers to providing bystander CPR in regions with low rates of bystander CPR and where OHCA was recognized in the emergency call	Retrospective cohort	Caller, Community/context	Targeted interventions for the community should include education in OHCA recognition, training in CPR as well as how to make an emergency call	
Park et al. 2018	South Korea	To evaluate whether the implementation of a bundle of three programs to improve OHCA outcomes	Before-after	System	Performance assessment through an individual dispatcher–targeted quality assurance protocol on T–CPR for every OHCA	
Lerner et al. 2019	Wisconsin (USA)	To describe the implementation of a novel centralized dispatcher CPR instruction program	Prospective before-after	System	A centralized EMCC to provide DA-CPR instructions decreases training, maintenance costs, and quality improvement efforts. The practice of giving CPR instructions is concentrated over a smaller number of dispatchers and the higher call volume leads to a higher level of experience	High rate of call transfers not related to OHCA (over-triage)
Lu et al. 2019	Taiwan	To evaluate DA-CPR in traumatic OHCA	Retrospective cohort *	Dispatcher, caller	Traumatic OHCAs have unique presentations and require a different approach regarding DA-CPR	Traumatic OHCAs have lower identification of cardiac arrest (6.3% versus 42.0%) and lower DA-CPR (5.4% versus 37.6%), when compared with medical OHCA
Kim et al. 2019	South Korea	To evaluate the bystander gender difference on successful DA-CPR	Cross-sectional *	Dispatcher		Less DA-CPR performed by female bystanders, less ROSC and favorable neurological outcome
Contri et al. 2019	Italy	To understand if sharing a detailed report on T-CPR activity to call-takers could improve their awareness on this topic and boost their performance	Retrospective cohort *	System	Sharing data about DA-CPR with dispatchers improves awareness and performance	
Kaneko et al. 2019	Japan	To compare the rate of B-CPR and PAD associated with DA-CPR	Retrospective cohort	Dispatcher	Dispatchers are trained and regularly update their skills in dispatch simulations and group discussions. DA-CPR was associated with significant increases in the rates of bystander CPR and PAD, and with significant improvements in neurological outcomes following OHCA	
Bagheri et al. 2019	Iran	To determine the effects of the implementation of the T-CPR program on the outcomes of OHCA	Before-after	Caller, community/context	After the implementation of a DA-CPR program, the number of successful CPR cases and survival rate increased; CPR-related complications decreased from 20% to 14.3%. It is recommended that dispatchers be trained in DA-CPR research	
Al Hasan et al. 2019	Kuwait	To measure the impact of DA-CPR on OHCA recognition, CPR rates and patient outcomes	Before-after	Dispatcher, system	Specific training (1-day intensive course), DA-CPR protocol (chest-compressions only), and quality assurance (audio recordings, feedback and performance evaluation) improve early OHCA recognition and CPR instruction rates	The lack of CPR public awareness, early defibrillation, and post-resuscitation care are some potential factors resulting in low OHCA survival to hospital discharge
Amen et al. 2020	USA	To evaluate racial and socioeconomic disparities in the provision of T-CPR instruction and subsequent CPR performance	Retrospective cohort	Caller, system	Increased likelihood of DA-CPR agreement based on increased income. No differences by race	Increasing patient age is associated with decreased odds of bystander agreement to perform CPR under dispatchers’ guidance
Zhang et al. 2020	China	To assess the process compliance, barriers and outcomes of OHCA in one of the earliest implemented DA-CPR programs	Retrospective	Caller, community/context	Efforts must be made to increase public awareness of OHCA, calling for help and competency in DA-CPR	Bystander barriers to DA-CPR included unstable emotional state, fear of harming the victim, fear of incorrectly performing CPR, and caller not being with the victim. Although the dispatcher explained the importance of DA-CPR, callers were unwilling to follow the instructions believing that only medical professionals could help the victim
Torossian et al. 2020	Michigan (USA)	1) To characterize OHCA bystanders’ demographics; 2) To understand the events from patient collapse until EMS arrival, with a focus on telecommunicator or DA-CPR (T-CPR); 3) Bystander knowledge/ training in CPR and their actions at the time of OHCA	Prospective observational *	Caller, community/context	CPR educational programs should prepare trainees to know that they may well respond to loved ones	CPR-trained bystanders who did not perform CPR identified panic, assessment barriers and physical reasons for not performing CPR
Sanko et al. 2020	California (USA)	To assess the impact of this new dispatch system on T-CPR	Before-after	Dispatcher	Improved T-CPR rates, increased rate of OHCA correctly identified, reduced time to OHCA recognition, and time to deliver the first chest compression with a specifically developed protocol (fewer questions, and elimination of questions that do not affect dispatch level of service or provision of emergency instructions)	
Kim et al. 2020	South Korea	To evaluate the effect of emergency call crowding on DA-CPR instruction performance in OHCA calls	Cross-sectional	System	Medical directors and stakeholders should examine the current crowding status of their dispatch center and design a strategic approach to reduce call crowding	An approximate 3% reduction in good neurological recovery was shown for every 30 s of delay in the detection of OHCA by EMCC dispatchers
Blewer et al. 2020	Singapore	To assess the cumulative effect of CPR-targeted public health interventions	Prospective cohort	Community/context	A bundle of three public health bystander-focused interventions (DA-CPR, a training program for CPR and AED, and a first responder mobile application) was associated with increased bystander CPR frequency and increased survival to hospital discharge after OHCA	
Fouche et al. 2020	Michigan (USA)	To explore the role of EMS in promoting timely bystander response during an OHCA event	Mixed-methods *	Dispatcher, community/context	1) Preparing the community for proactive bystander response through educational campaigns; 2) Facilitating bystander CPR during an event through DA-CPR and providing positive reinforcement to bystanders; 3) Reinforcing the importance of performing bystander CPR to the involved community after post-arrest care	
Lee et al. 2020	South Korea	To compare the real-world effects of audio-instructed DA-CPR and video-instructed DA-CPR	Retrospective cohort	Dispatcher	The dispatch center developed a protocol to change from an audio to a video call after one cycle of chest compressions; the video-call protocol was provided only when there were two or more bystanders at the scene and the caller had a mobile phone capable of video calls	Video calls delayed the time from the call to the start of chest compressions
Huang et al. 2020	Taiwan	To compare the effectiveness of DA-CPR instructed via landline calls, mobile calls, and landline calls transferred to mobiles	Prospective cohort	Caller	Transferring calls from a landline to a mobile is vital to improve OHCA recognition. Mobile callers have a higher possibility of being around the patient and are more willing to perform DA-CPR. The mobile enables the callers to utilize a speaker function. Calling from a mobile resolves the problem of distance and helps to monitor the compression frequency	Calls were delayed during the phone transfer for several reasons, including the caller searching for a mobile, forgetting the phone number, reporting a wrong number, being unfamiliar with phone operations, calling with a weak signal, and the dispatcher experiencing technical problems
Chen et al. 2020	China	To compare the provision and effectiveness of DA-CPR in rural and urban areas	Retrospective cohort	Community/context	DA-CPR rate was higher in urban areas than in rural areas, with a superior prognosis. Public health and education are warranted to encourage people to perform CPR and achieve higher bystander CPR rates, particularly in rural areas	
Balzanelli et al. 2020	Italy	To evaluate the efficacy in terms of ROSC and survival	Retrospective cohort	Dispatcher, caller	Compression-only DA-CPR in OHCA doubles the probability of ROSC and increase significantly the survival rate	
Pek et al. 2021	Singapore	To evaluate the impact of a DA-CPR program on B-CPR rate and outcomes of OHCA in a developing EMS system setting	Before-after	Dispatcher, system, community/context	A simplified DA-CPR program (1) a standardized DA-CPR protocol; 2) a standardized DA-CPR training package: 1-day DA-CPR workshop, didactic teaching, small group discussions, practical scenarios, and online training course; 3) a data collection tool to review dispatch audio recordings and assess performance; 4) a continuous quality improvement process; 5) a community CPR training program to increase public’s awareness on OHCA) can be successfully implemented in a developing EMS system and can contribute to higher B-CPR rate and improved OHCA survival	
Soon Lee et al. 2021	South Korea	To investigate whether video-instructed DA-CPR improved neurologic recovery and survival to discharge compared to audio-instructed DA-CPR in adult OHCA	Retrospective cohort	Dispatcher	Positive effects between the dispatcher and caller via video-instructed DA-CPR, such as real-time feedback, better identification of the patient’s condition, and better guidance when the caller was hesitant or panicked in the emergency situation	Video calls might not be possible when caller was not good at using a mobile phone and could not be connected to a video call; when the caller was confused and could not respond properly to the dispatcher’s instructions; when there was no other person in the vicinity other than one caller; and when caller was connected to a video call, it was difficult to transmit information on the site because proper operation such as camera switching was not possible
Hardeland et al. 2021	Scandinavia (Denmark, Norway, Sweden)	To describe OHCA call handling in EMCCs with a special focus on sensitivity of recognition of OHCA, provision of DA-CPR and time intervals	Observational multicenter	Dispatcher	Feedback, reassurance and quality assessment allow continuation of CPR until EMS arrival	Lack of CPR instructions to bystanders performing CPR before call indicates a knowledge gap. There is a need for further exploration of the consequences when dispatchers do not provide CPR instructions to a large group of bystanders in a group mostly excluded from studies on DA-CPR
Siman-Tov et al. 2021	Israel	To quantify and qualify DA-CPR (acceptance/rejection)	Retrospective cohort	Dispatcher, caller	DA-CPR dispatcher training should include skills for encouraging callers to overcome their natural resistance and tools to alleviate their distress, offering clear CPR instructions and providing ongoing encouragement and instruction. Dispatchers must be practicing EMTs or higher training, who undergo rigorous additional training involving approximately 150 h of theory and field studies. Reasons for caller's refusal to perform DA-CPR are varied; some reasons may be overcome with proper intervention; if DA-CPR is rejected/non-feasible by caller the dispatcher records the reason	
Riou et al. 2021	Australia	To explore the impact of caller perception of patient viability on initial recognition of OHCA by the dispatcher, rates of bystander CPR	Retrospective cohort	Dispatcher, caller	When calling the emergency number, saying that the patient is dead is the most direct way to describe OHCA in lay terms. Caller statements that the patient is already dead (one in five cases) are helpful for dispatchers to early recognize OHCA	A caller’s declaration of death significantly increased the likelihood of declining to perform DA-CPR
Wong et al. 2021	Singapore	To assess the effectiveness of community-level interventions (DA-CPR) and a mobile app to alert first responders	Retrospective cohort	Dispatcher, community/context	DA-CPR increased bystander CPR rates through a standardized dispatch protocol to recognize suspected OHCA through a systematic “no–no-go” process (two key questions: Is the patient conscious? Is the patient breathing normally?) The protocol guides the dispatcher on how to instruct the caller to commence CPR, limited to chest compression only	The role of apps in increasing bystander CPR is unclear
Tzeng et al. 2021	Taiwan	To investigate the association between DA-CPR performance and OHCA outcomes among communities with different socioeconomic status	Retrospective cohort	Caller	Strategies to promote public awareness of cardiac arrest should be tailored to neighborhoods with lower socio-economic status	Lower socio-economic status was associated with a longer time to dispatcher recognition of OHCA, lower rate of agreement in performing DA-CPR, and worse outcomes
Chocron et al. 2021	Washington (USA)	To evaluate the quality of bystander CPR and whether performance varied according to the number of bystanders or provision of T-CPR	Retrospective cohort	Dispatcher	Active coaching, background metronome or messages to prioritize compressions and emphasize “push hard, push fast, don’t stop” may be an effective strategy to influence bystander compression rate and performance	Additional bystander actions to rotate compressors or deliver an AED were uncommon, even when there were multiple rescuers
Nikolovski et al. 2021	Serbia	To analyze the influence of dispatcher assistance during CPR	Prospective observational	System	Education and prevention of EMCC dispatchers' burnout syndrome are excellent investments in saving people's lives. Improving the role of EMS dispatchers in the community and identification of bystanders’ potential fears through comprehensive educational campaigns for the general population could overcome those fears. Identification of the causes of bystanders’ fear of performing CPR improve DA-CPR, increase bystander cooperation, and improve results of bystander CPR	
Goto et al. 2021	Japan	To determine the optimal DA-CPR instructions for OHCA	Prospective observational	Dispatcher	Conventional DA-CPR was feasible in 105 of OHCA and was associated with better outcomes compared with compression-only DA-CPR. Collapse-to-initiation of DA-CPR time was shorter in the conventional DA-CPR than in the compression-only DA-CPR by approximately one minute, probably because bystanders were CPR trained	One concern in performing conventional DA-CPR is the time delay in initiating bystander CPR compared with compression-only DA-CPR when bystanders have not been trained in CPR before
Perera et al. 2021	Australia	To compare language barrier and non-language barrier OHCA call time intervals	Retrospective cohort	Dispatcher	DA-CPR protocols should be revised for handling OHCA calls with language barriers, rewording parts of the script with language barrier callers in mind. If the language can be identified, an interpreter could be engaged in a three-way call, even if this is a lengthy process that may exceed viability timeframes for CPR	Language barrier calls experienced longer delays to key points in the OHCA protocol. Two particularly problematic parts in language barrier cases were identified: address acquisition and positioning the patient for CPR. Communication protocols for these phases of the call could be reviewed in order to help minimize delays to OHCA recognition and telecommunicator CPR
Sanko et al. 2021	USA	To evaluate if the implementation of the Los Angeles-Tiered Dispatch System (LA-TDS) protocol was associated with improved T-performance among callers with limited English proficiency	Retrospective cohort	Dispatcher, caller	Simplified dispatch protocols may overcome language barriers (decreasing the number of questions, not asking questions that have already been answered, treating vague answers regarding life status, and offering early reassurance that help is on the way)	Call transfer to a translation service may have implications for the caller-dispatcher relationship
Sonh et al. 2022	South Korea	To evaluate the effects of bystander CPR and DA-CPR on outcomes after OHCA	Prospective observational	Dispatcher	The interaction between compression-only CPR and DA-CPR was significantly associated with good neurological and survival outcomes after OHCA, suggesting the use of 30:2 DA-CPR only in case of trained bystanders	
Fareed et al. 2022	Pakistan	To evaluate the acceptability of T-CPR by the bystanders and identify baseline quality measures of T-CPR	Cross-sectional	Dispatcher, community/context	In a developing setting, the results show high acceptability of DA-CPR by bystanders	Considerable delays in recognizing cardiac arrest and initiation of DA-CPR. Training for telecommunicators could reduce these delays
Guerrero et al. 2022	Singapore	We assessed the relationship of T-CPR process metrics with patient outcomes	Retrospective analysis	System	The measurement and adequate performance of DA-CPR metrics and participation in a cardiac arrest registry and centralized systems may improve DA-CPR provision and OHCA patients' outcomes	Barriers to CPR, when documented, were present in 32.3% of cases, the most common being the inability to move the patient to an appropriate position. Each additional second of delay to first compression was associated with a 0.8% decrease in the odds of survival
Lim et al. 2022	Singapore	To investigate the impact of COVID-19 on barriers to DA-CPR	Before-after	Caller, community/context		Increased changes in patient status during the pandemic; specifically, there were more cases where patients were reportedly conscious at the time of call but became unconscious thereafter. Specific barriers: more OHCA at home, less witnessed OHCA, reduction in mobility and first responders’ availability
Park et al. 2022	South Korea	To estimate the effect of a new DA-CPR support training program on the survival outcomes	Before-after	System, community/context	Quality assurance: EMCC medical directors are required to review about 10% of DA-CPR audio recordings and provide feedback to the dispatchers. A specifically developed program aim at enhancing compliance with DA-CPR, tailored for middle-aged housewives and elderly people. Cooperation with other associations is needed to provide CPR educational opportunities not only to trainees visiting the education center, but also to senior citizens visiting senior citizen centers	
Ushimoto et al. 2022	Japan	To investigate how large-scale earthquakes and tsunamis as well as subsequent nuclear pollution influenced B-CPR performance for OHCA witnessed by family and friends/colleagues	Retrospective cohort	Community/context	BLS training might serve as preparedness for disaster and major accidents	A large-scale disaster may influence bystander-initiated CPR and outcomes of OHCA witnessed by family/friends/colleagues
Ong et al. 2022	Asia–Pacific	To evaluate the impact of a DA-CPR program on B-CPR rates and survival	Before-after	Dispatcher, community/context	Implementing a DA-CPR program in countries with mature EMS systems and those with developing ones is feasible and impacts bystander CPR and favorable outcomes. The implementation program is based on standardized DA-CPR protocol and DA-CPR training workshop (1-day training workshop: didactic lectures, small group discussions, practical scenarios, online DA-CPR training program, recording review and performance evaluation), in addition to a public education program	
Goto et al. 2022	Japan	To determine optimal DA-CPR instructions for bystanders to perform CPR after pediatric OHCA	Retrospective cohort	Dispatcher	In case of pediatric OHCA, dispatcher-assisted conventional CPR (30:2) should be provided to trained bystanders and dispatcher-assisted compression-only CPR should be provided to untrained bystanders or trained bystanders unwilling to provide rescue breaths	
Ng et al. 2022	Singapore	To find the incidence of DA-CPR initiated for non-OHCA cases	Retrospective multicenter	Caller	Chest compressions initiated on patients not in cardiac arrest did not result in any reported complications and were not associated with in-hospital mortality. This provides reassurance for the continued implementation of DA-CPR	The false positive recognition rate of cardiac arrest by dispatchers was 40.0%. Of these, 52.7% progressed to DA-CPR
Wong et al. 2023	Hong Kong	To evaluate the impact of post-Dispatch Advice on bystander CPR + reasons for not providing DA-CPR	Retrospective	Dispatcher, caller, community/context	No–No-Go protocol—identification of cardiac arrest is enhanced by additional protocol-driven telephone interrogations: (1) regard no breathing if the breathing status cannot be provided by the caller at scene; (2) dispatchers are encouraged to use an ‘agonal breathing diagnostic tool’ whenever in doubt; (3) dispatchers are encouraged to stay online with the callers if the patient is categorized as unstable	48% of callers declined DA-CPR for not being physically with the victim. More public education is necessary to provide information about how to recognize cardiac arrest and what occurs in DA-CPR
Huang et al. 2023	Taiwan	To compare the implementation of DA-CPR in private homes and public places	Retrospective cohort *	Community/context	DA-CPR was more successfully performed in private homes than public places, although adequate emotional support to bystanders may further promote DA-CPR. The DA-CPR protocol should be modified for different locations (public vs private)	
Yacobis-Cervantes et al. 2023	Spain	To design an algorithm to improve the T-CPR response protocol	Not defined	Dispatcher	Dispatchers should be able to identify and recognize the caller’s emotions and adapt to them throughout the call to achieve better emotional control. Throughout the call, dispatchers should use various emotional control techniques, such as positive reinforcement, feedback, maintaining continuous verbal contact, and not engaging in provocations if initiated by the caller. Encourage the witness not to leave the victim alone and to continue the process even if the victim has high comorbidity	
Missel et al. 2023	Michigan (USA)	1) To evaluate how physical limitation related to the outcome of time required to reposition a patient to begin CPR, understanding that delays to early CPR adversely affect the chances of survival. 2) To evaluate how delay groups were associated with process outcomes of bystander CPR provision, and the clinical outcomes of survival and survival with good neurologic function	Retrospective cohort	Dispatcher	DA-CPR protocols should include the option of incorporating CPR “as-is” in the subset of patients where repositioning cannot be achieved in a timely manner	The challenge of repositioning produced an average 90-s delay compared to those without barriers
Binhotan et al. 2023	Saudi Arabia	To evaluate T-CPR performance	Retrospective cohort	System	Avoiding unnecessary call transfers could improve the time to first chest compression. Training dispatchers to provide DA-CPR do not require transferring OHCA calls to a physician. A quality improvement program for EMCC is recommended to improve DA-CPR performance	Transferring OHCA calls to a physician takes longer times
Chen et al. 2024	Taiwan	To compare different communication models in the dispatch center	Before-after	Dispatcher	A new protocol based on persuasive communication features (reciprocity, commitment, and consistency), widely used in marketing and advertisement, allowed for shorter OHCA recognition times and chest compression delivery. Additionally, although not statistically significant, the rate of DA-CPR increased from 54.1% to 7.8%	
Chen et al. 2024	Taiwan	To compare DA-CPR outcomes in private homes versus public places	Retrospective cohort	Community/context	DA-CPR had a greater acceptance rate in private homes than in public places (64.9% vs 47.7%) Family members in private homes had a higher proportion of chest compressions and shorter times to chest compression compared to non-family members	Despite having a higher rate of witnessed collapse, OHCA in public places had a lower rate of OHCA recognition and chest compressions
Huang et al. 2024	Taiwan	To examine the effects of implementing a dispatcher-assisted PAD protocol on DA-CPR	Before-after	Dispatcher	No significant difference was observed in the performance of dispatchers in terms of CPR instruction when adding to the protocol instructions on how to retrieve a public access defibrillator	
Riva et al. 2024	Sweden	To evaluate if compression-only DA-CPR by trained laypersons is non-inferior to standard DA-CPR in adult OHCA	Randomized controlled trial (pilot)	Dispatcher	Compression-only DA-CPR versus standard DA-CPR by trained laypersons was feasible, with no differences in safety measures or short-term survival	
Zhao et al. 2024	China	To evaluate the current status of care quality of pediatric OHCA in China	Prospective, multicenter, population-based registry study	Dispatcher	Dispatchers need to be proficient during pediatric OHCA, providing correct DA-CPR. instructions. Regular pediatric first aid training should be conducted for dispatchers to improve their response	The rate of DA-CPR in pediatric OHCA is low (10.9%)

PCC population (dispatcher and/or caller), concept (EMCC system), context (community/context) framework. Pros: key advantages or positive findings reported in the study regarding DA-CPR. Cons: limitations, challenges, or negative aspects highlighted in the study

*B-CPR* bystander cardiopulmonary resuscitation, *BLS* basic life support, *CPR* cardiopulmonary resuscitation, *DA-CPR* dispatcher-assisted cardiopulmonary resuscitation, *EMCC* emergency medical communication center, *EMS* emergency medical services, *OHCA* out-of-hospital cardiac arrest, *PAD* public access defibrillation, *PCC* population, concept, context, *ROSC* Return of spontaneous circulation, *T-CPR* telephone-cardiopulmonary resuscitation. *Conference abstracts

### Results of individual sources of evidence

**Fig. 2:**
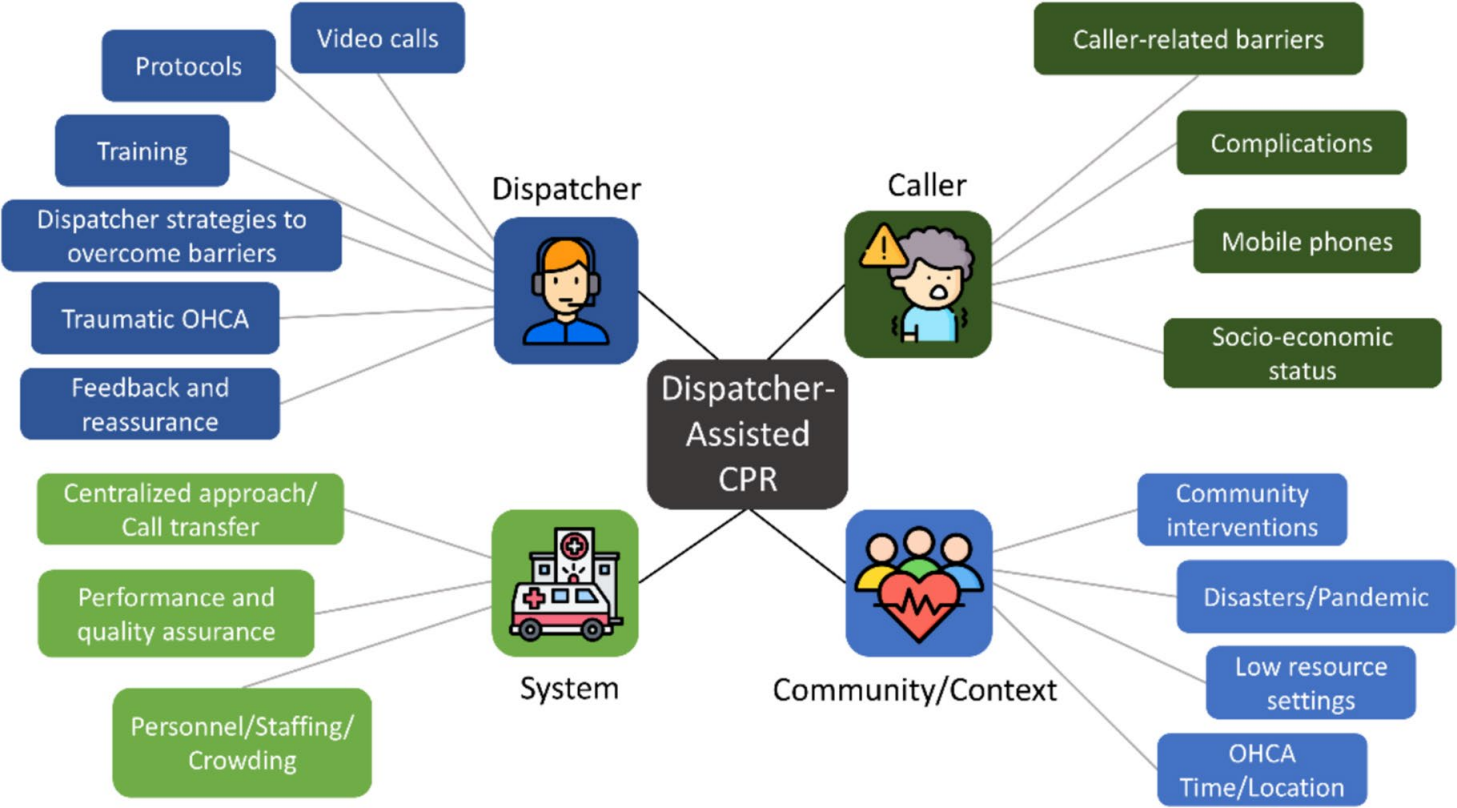
Main categories and subcategories of included studies. *CPR* cardiopulmonary resuscitation, *OHCA* out-of-hospital cardiac arrest. Icon credits: www.flaticon.com

The studies were grouped into four main categories: dispatcher, caller, system, and community/context, with 17 subcategories (Fig. 2). Thirty-three studies focused on dispatchers [10, 11, 16, 18, 35, 41–43, 47–49, 51, 52, 54, 55, 57, 59, 61, 62, 65, 68, 71, 72, 74–77, 79, 82, 83, 85–87], 17 on callers [10, 11, 36, 38, 45, 53, 54, 56, 63, 67, 70, 76–79, 84, 86], 14 on the EMCC system [17, 35, 44, 50, 51, 58–60, 69, 70, 73, 77, 80, 81], and 19 on the community/context [11, 18, 36–40, 46, 53, 57–59, 64–67, 72, 78, 84].

#### Dispatcher

##### Protocols

Fifteen studies addressed DA-CPR protocols [11, 35, 42, 43, 47, 49, 51, 55, 59, 61, 65, 71, 75, 79, 83]. Delayed OHCA detection was linked to a 3% decrease in good neurological recovery for every 30 s of delay [51]. Early symptoms (e.g. agonal breathing or seizures) can hinder CA identification, delaying CPR. Implementing specific breathing assessment tools or targeted questions may enhance OHCA recognition [11, 75]. Protocols with fewer questions reduce the time to confirm CA and initiate chest compressions [11, 51, 55]. A simplified algorithm, based on the 2010 International Liaison Committee on Resuscitation (ILCOR) recommendations, omitting the look-listen-feel assessment and focusing on chest compression-only CPR, achieved higher DA-CPR rates [51, 55, 59, 65].

Four studies compared dispatcher-assisted conventional CPR (30:2 ratio of compressions to breaths) with compression-only CPR, yielding mixed results [42, 43, 61, 79]. A randomized controlled trial pilot study comparing compression-only DA-CPR versus standard DA-CPR by trained laypersons showed no differences in safety measures or short-term survival [83]. Conventional DA-CPR showed better outcomes in adults and pediatrics [42, 43], but was feasible in only 10% of cases [43]. Instructions for rescue breaths may further delay CPR [42]. Goto et al. reported shorter collapse-to-CPR times with conventional DA-CPR, likely due to CPR-trained bystanders [42]. Protocols including compression-only CPR enhance DA-CPR rates even in settings where CPR public awareness is scarce [35]. Providing additional information on public Automated External Defibrillator (AED) retrieval did not affect DA-CPR performance [47].

Dispatchers are encouraged to stay online not only during DA-CPR, but also for unstable patients [11, 65]. Though rare even with multiple rescuers, actions like rotating compressors or using an AED should be encouraged for CPR-trained bystanders [71]. Notably, lower DA-CPR rates and reduced survival outcomes were observed in female bystanders, suggesting the need for tailored approaches based on bystander gender [49].

##### Feedback and reassurance

Active coaching and encouraging phrases like “keep going”, “you’re doing great,” and “the ambulance is on its way” can motivate bystanders [16]. Performance assessments, used in 74–80% of OHCA calls [16], may enhance CPR quality by influencing compression rate, providing verbal cues (e.g. "push hard", "push fast", "don't stop"), or acting as a background metronome [16, 71, 72].

##### Dispatcher strategies to overcome barriers

Language barriers increased delays in emergency calls, adding 15 s for address acquisition, 18 s for CA recognition, and 44 s for DA-CPR initiation [85]. Multilingual interpreting services via three-way calls represent a potential solution but can be time-consuming and affect caller–dispatcher relationships [76, 85]. Dispatcher protocols should address language barriers by simplifying scripts, reducing or rephrasing questions, and offering early reassurance that help is on the way [76, 85]. In addition, Riou et al. found that using terms of futurity (“going to, will”) or obligation (“need, have to”) led to high caller agreement (97% and 84%, respectively), while terms of willingness (e.g., “want, would like”) resulted in low agreement (43%) [87].

In about 20% of CA calls, callers stated the patient was already dead, using words like “dead”, “passed away”, “deceased”, or similar. Such declarations increased DA-CPR refusal rates (38% vs. 10%, adjusted OR 4.59, 95% CI [2.49–8.52], p < 0.001), yet 15% of these patients achieved return of spontaneous circulation [86]. Caller statements about death can help dispatchers identify CA quickly but should not discourage them from providing CPR instructions.

Caller panic can hinder effective collaboration, crucial for DA-CPR [82]. Dispatchers should recognize and adapt to bystanders’ emotions, maintaining control through techniques like positive reinforcement, motivation, emotional support, feedback, continuous verbal contact, and avoiding provocation. Persuasive communication strategies (reciprocity, commitment, and consistency), widely used in marketing and advertisement, enhance DA-CPR rate [41]. Witnesses should also be encouraged to stay with the victim [10, 82]. Repositioning the patient is a common barrier, causing delays of up to 90 s [74, 77]. Strategies to improve resuscitation rates should address logistical challenges, including allowing CPR “as-is” when physical barriers are present [74, 77].

##### Training

Seven studies discussed dispatchers’ training [10, 18, 35, 48, 57, 59, 68]. A bundled approach combining dispatcher training, quality assurance, and community education may impact DA-CPR rates and OHCA outcomes [35, 57]. Even in resource-limited settings, training telecommunicators can help reduce delays in CA recognition and CPR initiation [18].

Candidates for dispatcher roles should be experienced EMS providers, undergo up to 150 h of additional training, pass a specific test, and have their fieldwork monitored by a supervisor [10]. Regular skill updates through simulations and group discussions are essential [48]. Training can include an intensive one-day workshop with lectures, group discussions, and practical scenarios, supported by an online DA-CPR program. A more comprehensive approach may involve quality assessments, reviewing real calls, structured data collection, and feedback to EMCC providers [35, 57]. A standardized one-day workshop and online training course have proven feasible, even in developing countries [59]. Moreover, considering the low occurrence of pediatric OHCA, dispatchers should be regularly trained in pediatric Basic Life Support (BLS) to improve their response when providing DA-CPR [68].

##### Traumatic OHCA

A single study examined DA-CPR in traumatic OHCA [61], reporting lower CA identification (6.3% vs 42.0%) and DA-CPR rates (5.4% vs 37.6%) when compared to nontraumatic OHCA; the main reasons included callers not being with the victim, third-party callers, and unsafe scenes. These results suggest that traumatic OHCA requires tailored approaches in DA-CPR protocols [54].

##### Video calls

The widespread availability of smartphones has enabled EMCCs to use video calls, improving rescuer compliance, DA-CPR rates, and OHCA outcomes [52, 62]. Video calls provide call-takers with a visual patient assessment, allowing for focused instructions, real-time feedback, and better guidance in cases of panic or hesitation [52, 62]. However, challenges include callers struggling with phone functionality, older devices, or poor network connectivity [62]. While video calls may slightly delay CPR initiation, the potential for high-quality, supervised BLS justifies their use [10]. Studies recommend using video calls only when two or more bystanders are present, with the phone positioned to show both the patient and rescuer [10, 52]. The Seoul dispatch center implemented a protocol initiating video calls after one cycle of chest compressions [52].

#### Caller

##### Caller-related barriers

Eleven studies reported caller barriers [10, 11, 53, 54, 67, 76–79, 84, 86], with DA-CPR refusal rates ranging from 23.5% to 50.3% [10, 11]. Case et al. categorized barriers into personal factors (physical or emotional limitations), CPR knowledge (skill deficits, perceived benefits), and procedural issues (communication, language barriers, delayed arrest recognition) [84]. Common barriers included unstable or panicked emotions [10, 67, 77–79], call disconnection [67], inability to reposition the victim [10, 11, 77], and fear of harming the victim, even in traumatic OHCA cases [54, 67, 79]. Notably, panic, assessment challenges, and physical limitations prevented CPR in 25.8% of cases, even among trained bystanders [78].

Third-party calls, where the caller is not at the OHCA scene, occurred in up to 48% of cases [10, 11]. Another barrier was signs of death, with callers deeming DA-CPR unnecessary due to the victim's age or health conditions [10, 11, 86].

Limited language proficiency, affecting 14.2% of calls in an Australian study [85], often required repeated questions, particularly for address acquisition and patient positioning for CPR [76]. As previously reported, language-related barriers could be addressed by dispatchers with specific strategies.

Lim et al. reported a higher rate of barriers to DA-CPR during the COVID-19 pandemic (60% vs 50.5% pre-pandemic), primarily due to changes in the victim's status (e.g., initially conscious but later unconscious) [53]. This may stem from rapid patient deterioration due to severe respiratory failure, difficult recognition of respiratory symptoms, or delayed EMS response. Notably, fear of COVID-19 transmission accounted for only 0.5% of barriers, and no bystanders refused to perform DA-CPR [53].

##### Complications

Overestimating caller-provided information can result in DA-CPR being performed on patients not in CA; this occurred in 52.7% of cases in one study, but no compression-related complications were reported in hospital records [56]. Furthermore, implementing a DA-CPR program reduced CPR-related complications, such as rib fractures, from 20% preimplementation to 14.3%, demonstrating that the benefits of DA-CPR outweigh the minimal risk of injuries [36].

##### Mobile phones

A study compared DA-CPR provided via landline and mobile phones. Mobile phones offered advantages, allowing callers to stay near the victim and reducing call-to-compression time. However, challenges included unfamiliarity with device functions (e.g., using the speaker or camera), technical issues, and low signal strength [45].

##### Socio-economic status

Higher-income and wealth correlated with increased DA-CPR agreement, likely due to higher education, greater CPR training opportunities, earlier CPR education, and better health literacy [38, 70]. Conversely, lower socio-economic status was linked to delayed OHCA recognition, lower DA-CPR agreement, and poorer outcomes [63]. These results highlight the need for tailored DA-CPR programs and CPR training strategies targeting lower socio-economic groups to enhance compliance and effectiveness.

#### System

##### Centralized approach/call transfer

Two studies highlighted improved performance and outcomes centralizing CA calls to specific EMCCs [60, 73]. Lerner et al. reported a 20% increase in chest compression delivery and an 8% rise in survival for CA calls handled by centralized EMCCs [73]. Ro et al. observed higher DA-CPR rates (adjusted OR 4.57, 95% CI [3.26–6.42]) and increased survival to discharge (adjusted OR 1.26, 95% CI [1.01–1.5]) after centralization [60].

Centralized DA-CPR offers benefits like reduced costs and efforts for call-taker training and quality assurance [73], as well as increased expertise due to higher call volumes [60, 73]. However, transferring many non-CA calls risks over-triage and EMCC crowding [73]. Operational barriers include failure to transfer CA calls from primary public answering points to second-level EMCCs equipped for DA-CPR [70, 77]. In addition, transferring CA calls to a physician for CPR instructions caused a median delay of 231.5 s in starting compressions. DA-CPR training for all dispatchers could eliminate unnecessary call transfers [17].

##### Performance and quality assurance

Six studies addressed performance review of DA-CPR calls [35, 44, 58, 59, 69, 80]. Structured reviews of call recordings are part of bundled interventions to enhance DA-CPR, [35, 59, 69]. Sharing data with EMCC providers, including patient outcomes [59], improves CA recognition (+ 7%) and DA-CPR rates (+ 13%) [80]. Approaches vary: Park et al. described monthly individual dispatcher evaluations for every OHCA [69], while others involved reviewing around 10% of call recordings by dispatch medical directors to provide feedback [58]. Data collection often uses standardized tools [59], mainly based on the American Heart Association (AHA) policy recommendations [15, 44, 88]. However, meeting AHA goals may be challenging, even for high-performing EMCCs, and requires significant resources [44].

##### Personnel/staffing/crowding

Two studies examined the effects of high call volumes on DA-CPR [50, 51]. EMCC crowding due to increased hourly call volumes led to delays in DA-CPR initiation [50]. large call volumes made distinguishing OHCA from non-OHCA calls more challenging, causing delays in CA identification, call transfers, and DA-CPR initiation [51]. In addition, preventing dispatcher burnout, alongside proper training, could improve working conditions and enhance preparedness for DA-CPR processes [81].

#### Community/context

##### Community interventions

Nine studies highlighted that a bundled approach improves DA-CPR agreement [11, 38, 58, 59, 65, 67, 72, 78, 84], reinforcing the "System Saving Lives" concept [4]. Implementing DA-CPR programs should include community interventions to raise CA awareness and educate the public on their role during emergency calls [11, 59, 65]. Public education should focus on identifying OHCA, performing CPR, and effectively interacting with EMCCs [37, 84]. A well-prepared population can expedite OHCA recognition, reduce CPR initiation times, and improve survival [67]. Reinforcing the importance of B-CPR within the community after post-CA care, sharing positive experiences, can enhance collaboration between EMCCs and the public [72].

CPR training should emphasize that emergencies may involve loved ones [78] and target middle-aged housewives and elderly individuals, common witnesses of CAs, with hands-on CPR and mobile phone use training (e.g., speaker mode activation) [58]. Collaboration between EMS, local associations, and senior centers is vital for achieving these goals. In addition, tailored DA-CPR programs and training for lower socio-economic groups are essential to improve compliance and ensure effective CPR delivery, particularly in pediatric OHCAs in underserved communities [38].

##### Disasters/pandemic

Large-scale disasters can impact DA-CPR compliance and OHCA outcomes. Reduced B-CPR rates may result from decreased social participation and psychological reactions, such as the fear of nuclear pollution observed in Japan in 2011, which discouraged performing CPR on strangers [64]. Although the COVID-19 pandemic did not affect laypeople’s willingness to perform DA-CPR, movement restrictions increased home OHCAs and temporarily disrupted first responder activities [53]. Community training in BLS and AED use can enhance both community engagement and disaster preparedness [53].

##### Low resource settings

Three studies demonstrated the feasibility of implementing DA-CPR programs in low-resource countries [18, 36, 57]. The Pan-Asian Resuscitation Outcomes Study found that a comprehensive approach, including dispatcher training, EMCC quality assurance, and public education, significantly improved DA-CPR provision [57]. In Iran, dispatcher training increased OHCA survival from 56.5% to 72.4%, with more patients showing good neurological outcomes [36]. Ahmed et al. reported high DA-CPR acceptance (95.4%) in Pakistan but noted that CPR initiation exceeded the AHA-recommended three-minute window in 91.3% of cases [18]. These results suggest areas for improvement, particularly in OHCA recognition and CPR training for the general population.

##### OHCA time/location

Urban areas showed higher DA-CPR acceptance than rural areas (74.9% vs 67.7%), with better outcomes [39]. Family members witnessing OHCA at night demonstrated good compliance but faced challenges in CA recognition and resuscitation performance [66]. Nighttime and private settings limit bystander intervention and AED access [66]. Similarly, DA-CPR acceptance and performance were higher in private homes as compared to public places [40, 46]. Callers in public settings were more likely to fail in recognizing abnormal consciousness or breathing, often due to environmental hazards, physical problems, or reluctance to follow dispatchers’ instructions; conversely, emotional and psychological barriers were encountered less frequently in these settings [40]. DA-CPR instructions should be tailored to account for location-specific challenges [40, 46].

## Discussion

This scoping review identified a series of factors influencing the provision and effectiveness of DA-CPR, in response to the knowledge gaps outlined in the 2019 ILCOR Recommendations. These gaps included limited evidence on dispatcher background and training, the optimal sequence for CPR instructions, the components of effective quality improvement programs, and the integration of emerging technologies, such as video calls and multilingual support systems [24]. Aldridge et al. identified a range of psychological, physical, and communication barriers that hamper DA-CPR, highlighting the necessity for implementation strategies that address these challenges in practice [9]. The scoping review by Dainty et al. and the recent 2024 ILCOR CoSTR reported that many proposed interventions to optimize DA-CPR were assessed in simulated studies, lacking high-quality evaluation in real-life settings [25, 26].

By focusing exclusively on real-world OHCA events, this scoping review contributes to a more accurate understanding of DA-CPR practices in operational settings, emphasizing organizational and contextual elements related to DA-CPR implementation. The results align with the Systems Saving Lives framework, demonstrating that a multifaceted approach is paramount for improving bystander response rates and OHCA outcomes [4]. The four main categories used to categorize our results (dispatcher, caller, system, and community/context) reinforce the concept of a collaborative interconnection between healthcare systems and communities. Notably, most of the included studies were related to dispatchers and callers, underscoring that the interaction between these two actors plays a vital role in OHCA response [89].

Dispatcher-related interventions, including simplified protocols and reduced questions, emerged as cardinal in improving caller compliance with DA-CPR and reducing delays in CA recognition and CPR initiation [11, 51, 55, 59, 75]. To overcome difficulties, barriers, and time delays, DA-CPR protocols should consider compression-only instructions, providing conventional CPR only to trained bystanders [42, 43, 61]. Compression-only DA-CPR is easier to teach over the phone and may represent a viable option for starting a DA-CPR program, for low-resource settings, or when community CPR awareness is low [35]. Results from a pilot study comparing telephone-assisted compression-only and standard CPR reported no differences in short-term survival [83]. The interaction between EMCC providers and laypeople plays a fundamental role, underscoring the need for tailored communication strategies 89. Dispatchers should acquire communication skills and apply coaching techniques [16, 71, 72], aimed at overcoming caller-related barriers, such as emotional distress, language issues, and misconceptions about resuscitation [10, 76, 82, 85–87]. Dispatchers’ training may include different strategies, ranging from online courses to workshops, didactic lectures, and practical scenarios [57, 59]. A more comprehensive approach could include regularly scheduled meetings with simulations, review of real calls, group discussions, and a wider quality assessment program [48, 57].

In recent years, video calls emerged as an effective support tool, allowing for better CA identification and real-time feedback during DA-CPR, improving CPR quality [19, 20]. Our findings confirm these advantages and, on the other hand, highlight a series of potential problems, suggesting that they should be used when two or more bystanders are present [10, 52]. Their use should be pondered against their limits, such as delays in starting a video call, inability to activate video functions, or other technical problems [10, 45, 52, 62].

Caller-related factors, such as emotional distress or language barriers, significantly impact an effective DA-CPR provision, preventing callers' ability to follow dispatcher instructions [9–11, 67, 78]. However, some reasons for callers’ refusal to perform DA-CPR may be overcome with proper intervention [10]. Language barriers can delay CA identification and the initiation of CPR, emphasizing the need for simplified instructions, including multilingual scripts and access to interpreter services [76, 85]. These challenges underscore the importance of tailored communication strategies and culturally sensitive protocols to improve caller compliance and obtain collaborative interaction [89].

System-level interventions demonstrated significant potential to enhance DA-CPR performance by streamlining emergency call handling and standardizing protocols. Centralized systems often result in greater dispatcher expertise and efficiency [25, 60, 73], while structured performance evaluations allow for continuous quality improvement [15, 59, 69, 80].

Community-based projects were associated with improved bystander engagement in CPR. Public education campaigns and targeted training programs, particularly in specific groups, such as middle-aged housewives or elderly individuals who are often present during OHCAs, were shown to increase bystander participation during emergencies [58, 78]. In addition, collaborations between EMCCs and local organizations, such as schools, community centers, and senior groups, play a key role in understanding the role of dispatchers, thus strengthening a collaborative interaction between citizens and EMS [11, 58, 59].

Dispatcher-assisted CPR represents a vital link in the chain of survival, with significant potential for improving outcomes. Its effectiveness can be enhanced by tailoring strategies to different cultural contexts, a need that becomes even more pressing in multicultural or multiethnic societies. Differences in the organization of EMS across countries significantly influence the implementation and effectiveness of DA-CPR. High-resource countries often benefit from centralized dispatch centers, standardized protocols, structured training programs, and integrated quality assurance systems [90–93], while low-resource settings may face challenges related to staffing, limited infrastructures, and scarce community awareness [94]. Despite these differences, studies from various geographic contexts demonstrate that context-adapted approaches, even with limited resources [18, 36, 57], can improve DA-CPR performance when supported by targeted training and community education programs. Future research should explore adjustable communication protocols that respect cultural sensitivities and prioritize practical solutions to mitigate caller panic and emotional distress while maintaining clinical efficacy. Additionally, there is a need for actionable solutions to overcome the challenges of DA-CPR in low-resource settings, leveraging community-based education or simplified, cost-effective technologies, to bridge resource gaps.

Lastly, artificial intelligence (AI) integration, particularly for adaptive, real-time DA-CPR guidance, presents an exciting frontier. Adaptive AI-driven support systems, like the Advanced Voice Interaction feature, may offer real-time emotional guidance and address linguistic or cultural barriers through multilingual capabilities [95]. Testing such innovations in real CA scenarios, especially in noisy environments or with multiple bystanders, will be decisive in validating their reliability and improving accessibility, thus providing real-time feedback and data collection.

## Strengths and limitations

The strength of this scoping review is its analysis of real-world DA-CPR implementation, providing evidence from actual clinical practice rather than simulations. This approach has identified concrete challenges and solutions in DA-CPR protocols. Notable limitations should be acknowledged. The retrospective observational design of most studies limited the robustness of evidence and precludes direct causal relationships. Despite a comprehensive search, terminological variations may have led to the exclusion of relevant studies due to the lack of standardized terminology. To ensure the inclusion of the most recent and relevant evidence, the original study period reported in the protocol was extended to include studies published in 2024. Finally, the selected timeframe (2018–2024) may have excluded earlier studies of potential relevance, such as those included in the systematic review by Nikolaou et al.[21]; however, this decision was made to focus on real-world experiences published after the 2015 resuscitation guidelines [31] and to address the knowledge gaps highlighted in the 2019 ILCOR Recommendations [24].

## Conclusion

This scoping review mapped and synthesized existing evidence on DA-CPR, identifying approaches and contextual factors that may support its provision and strengthen bystander response in OHCA scenarios. The results emphasize the importance of tailored dispatcher protocols, effective training, and communication to overcome caller-related barriers like emotional distress and language challenges. System-level interventions, such as centralized EMCCs and quality assurance programs, associated with CPR training projects for the community, may further improve collaboration between citizens and EMCCs. Future research should move beyond descriptive studies and focus on evaluating the real-world impact of these strategies, providing a deeper comprehension of the effectiveness of the different DA-CPR approaches to improve OHCA response.

## Supplementary Information

Below is the link to the electronic supplementary material.

Supplementary file1 (DOCX 17 KB)

Supplementary file2 (PDF 102 KB)

## Data Availability

This scoping review is based on data from publicly available research studies and publications. As the review synthesizes data from existing literature, no new datasets were generated or analyzed during this study, and are associated with this work.
