# Supplementary material for: Experiences, challenges, and best practices of dispatcher-assisted cardiopulmonary resuscitation: a scoping review
Source: Intern Emerg Med. 2025 Aug 11;20(6):1869–900. doi: 10.1007/s11739-025-03991-7 (PMC12476418; doi:10.1007/s11739-025-03991-7)
Supplement: Supplementary file 1 — Supplementary file1 (DOCX 17 KB) [file 11739_2025_3991_MOESM1_ESM.docx]

**Challenges and best practices of dispatcher-assisted cardiopulmonary resuscitation: a scoping review – Imbriaco et al.**

Supplementary material 2. Database search strings

| Database | Search string |
| --- | --- |
| **Medline (PubMed)** | ((cardiac arrest) OR (out of hospital cardiac arrest) OR (OHCA) OR (sudden death) OR (sudden cardiac death))  AND  ((pre-arrival instruction*) OR ("audio-delivered"[All Fields] AND "instruction*"[All Fields]) OR (video-delivered instruction*) OR (video-assisted bystander) OR (telephone CPR) OR (telephone cardiopulmonary resuscitation) OR (telephone-basic life support) OR (dispatcher-assisted) OR (dispatcher-assisted bystander) OR (dispatcher-assisted cardiopulmonary resuscitation) OR (dispatcher-assisted CPR) OR (telecommunicator cardiopulmonary resuscitation) OR (telecommunicator CPR))  AND  ((survival) OR (hospital discharge) OR (return of spontaneous circulation) OR (ROSC) OR (neurological outcome) OR (cerebral performance category) OR (CPC))  Filters: Humans, from 2018/1/1 - 2024/12/31 |
| **CINAHL - Cumulative Index to Nursing and Allied Health Literature** | cardiac arrest OR cardiopulmonary resuscitation OR cpr OR resuscitation OR heart arrest OR out of hospital cardiac arrest OR ohca OR pre-hospital cardiac arrest OR pre-hospital heart arrest OR sudden cardiac death OR basic life support  AND  pre-arrival instruction OR audio-delivered instruction OR video-delivered instruction OR video-assisted bystander OR telephone cpr OR T-CPR OR telephone cardiopulmonary resuscitation OR Telephone-Basic Life Support OR Dispatcher-Assisted Bystander OR Telecommunicator Cardiopulmonary Resuscitation OR dispatcher-assisted cpr OR dispatcher-assisted cardiopulmonary resuscitation  AND  survival OR survival rate OR hospital discharge OR return of spontaneous circulation or rosc OR neurological outcome OR functional outcome OR favourable outcome OR neurological outcome post cardiac arrest OR cerebral performance category OR CPC  Filters: Human, from 01/01/2018 – 31/12/2024 |
| **EMBASE - Excerpta Medica dataBASE** | (cardiac arrest OR out of hospital cardiac arrest OR OHCA OR sudden death OR cardiopulmonary resuscitation OR CPR OR basic life support OR cardio-pulmonary resuscitation)  AND  (pre-arrival instruction OR audio-delivered instruction OR video-delivered instruction OR video-assisted bystander OR telephone CPR OR T-CPR OR telephone cardiopulmonary resuscitation OR dispatcher-assisted bystander OR telecommunicator cardiopulmonary resuscitation OR dispatcher-assisted OR dispatch)  AND  (survival OR hospital discharge OR return of spontaneous circulation OR ROSC OR neurological outcome OR cerebral performance category OR CPC)  Filter: from 01/01/2018 – 31/12/2024 |
| **Cochrane Library** | (cardiac arrest OR out of hospital cardiac arrest OR OHCA OR sudden death OR cardiopulmonary resuscitation OR CPR OR basic life support OR cardio-pulmonary resuscitation)  AND  (pre-arrival instruction OR audio-delivered instruction OR video-delivered instruction OR video-assisted bystander OR telephone CPR OR T-CPR OR telephone cardiopulmonary resuscitation OR dispatcher-assisted bystander OR telecommunicator cardiopulmonary resuscitation OR dispatcher-assisted OR dispatch)  AND  (survival OR hospital discharge OR return of spontaneous circulation OR ROSC OR neurological outcome OR cerebral performance category OR CPC)  Filter: from 01/01/2018 – 31/12/2024 |
| **Scopus (for grey literature – conference procedings)** | TITLE-ABS-KEY (cardiac arrest OR out of hospital cardiac arrest OR OHCA OR sudden death OR cardiopulmonary resuscitation OR CPR OR basic life support OR Cardio-Pulmonary Resuscitation) AND (Pre-arrival instruction OR audio-delivered instruction OR video-delivered instruction OR Video-assisted bystander OR telephone CPR OR T-CPR OR telephone cardiopulmonary resuscitation OR Telephone-Basic Life Support OR Dispatcher-Assisted Bystander OR Telecommunicator Cardiopulmonary Resuscitation OR dispatcher-assisted OR Dispatch) AND (Survival OR Hospital discharge OR return of spontaneous circulation OR ROSC OR Neurological outcome OR cerebral performance category OR CPC) AND PUBYEAR > 2017 AND PUBYEAR < 2025 AND ( LIMIT-TO ( DOCTYPE , "cp" ) ) |
